# Oxidized phosphatidylcholines are produced in renal ischemia reperfusion injury

**DOI:** 10.1371/journal.pone.0195172

**Published:** 2018-04-23

**Authors:** Zahra Solati, Andrea L. Edel, Yue Shang, Karmin O, Amir Ravandi

**Affiliations:** 1 Institute of Cardiovascular Sciences, St. Boniface Hospital Research Centre, University of Manitoba, Winnipeg, Manitoba, Canada; 2 Department of Physiology and Pathophysiology, University of Manitoba, Winnipeg, Manitoba, Canada; 3 Department of Animal Science, University of Manitoba, Winnipeg, Manitoba, Canada; The University of Tokyo, JAPAN

## Abstract

**Background:**

The aim of this study was to determine the individual oxidized phosphatidylcholine (OxPC) molecules generated during renal ischemia/ reperfusion (I/R) injury.

**Methods:**

Kidney ischemia was induced in male Sprague–Dawley rats by clamping the left renal pedicle for 45 min followed by reperfusion for either 6h or 24h. Kidney tissue was subjected to lipid extraction. Phospholipids and OxPC species were identified and quantitated using liquid chromatography coupled to electrospray ionization tandem mass spectrometry using internal standards.

**Result:**

We identified fifty-five distinct OxPC in rat kidney following I/R injury. These included a variety of fragmented (aldehyde and carboxylic acid containing species) and non-fragmented products. 1-stearoyl-2-linoleoyl-phosphatidylcholine (SLPC-OH), which is a non-fragmented OxPC and 1-palmitoyl-2-azelaoyl-sn-glycero-3-phosphocholine (PAzPC), which is a fragmented OxPC, were the most abundant OxPC species after 6h and 24 h I/R respectively. Total fragmented aldehyde OxPC were significantly higher in 6h and 24h I/R groups compared to sham operated groups (*P* = 0.03, 0.001 respectively). Moreover, levels of aldehyde OxPC at 24h I/R were significantly greater than those in 6h I/R (*P* = 0.007). Fragmented carboxylic acid increased significantly in 24h I/R group compared with sham and 6h I/R groups (*P* = 0.001, 0.001). Moreover, levels of fragmented OxPC were significantly correlated with creatinine levels (r = 0.885, *P* = 0.001). Among non-fragmented OxPC, only isoprostanes were elevated significantly in 6h I/R group compared with sham group but not in 24h I/R group (*P* = 0.01). No significant changes were observed in other non-fragmented OxPC including long chain products and terminal furans.

**Conclusion:**

We have shown for the first time that bioactive OxPC species are produced in renal I/R and their levels increase with increasing time of reperfusion in a kidney model of I/R and correlate with severity of I/R injury. Given the pathological activity of fragmented OxPCs, therapies focused on their reduction may be a mechanism to attenuate renal I/R injury.

## Introduction

Renal ischemia/reperfusion (I/R) injury is the leading cause of acute kidney injury (AKI) affecting 13.3 million patients every year [1]. AKI is considered to be the main risk factor for acute renal failure in conditions such as kidney transplantation, renal surgery, contrast media-induced nephropathy, sepsis, aortic bypass surgery and cardiopulmonary surgery [2]. It is accompanied by rapid kidney dysfunction resulting in an abrupt increase in plasma creatinine as a result of kidney injury. The exact mechanisms underlying renal injury are complex and yet to be fully understood. Reduced supply of blood during renal ischemia leads to decreased oxygen in peripheral tissue, depletion of ATP and activation of enzymes such as phospholipases and protease [3]. However, during reperfusion there is a rapid influx of blood and oxygen to the injury site that is accompanied by robust inflammatory and oxidative stress responses resulting in abrupt kidney dysfunction [1]. Previous evidence has shown that renal I/R injury is a final consequence of various pathological events including inflammation, oxidative stress, apoptosis and fibrosis [4].

One of the most abundant membrane phospholipids in renal tissue is phosphatidylcholine (PC). Aside from being a part of the membrane bilayer, membrane phospholipids (PL) have roles in energy storage and cell signaling [5]. Previous evidence also demonstrated that phospholipids (PL) such as phosphatidylethanolamine (PE), PC, and phosphatidylinositol (PI) are potential markers of kidney disease [6].

Oxidative stress is the key meditator of I/R injury due to the inability of the innate antioxidant defense system to buffer the large burst of free radicals, which ultimately results in membrane lipid peroxidation [4]. Polyunsaturated fatty acids in the Sn-2 position of membrane PLs can be oxidized by reactive oxygen species (ROS), forming heterogeneous pools of end products, particularly fragmented and non-fragmented oxidized phospholipids (OxPLs) [7]. Fragmented OxPCs are biologically active compounds that can be recognized by the innate immune system through interaction with Toll-like receptors [8, 9], scavenger receptors [10–12], and natural antibodies [13, 14]. They have also been implicated in many disease states such as atherosclerosis and calcific aortic valve stenosis [15, 16]. OxPL participate in the pathophysiology of atherosclerosis [17] via induction of vascular inflammation [18] and apoptosis in foam cells [19]. In addition, patients with peripheral artery disease [20], and acute coronary syndromes [21] have elevated OxPL plasma levels. Epidemiological studies also reveal that OxPC can predict the occurrence of cardiac death, myocardial infarction (MI) and stroke [22–24]. We have recently shown that during cardiomyocyte I/R injury, there is a significant increase in fragmented oxidized phosphatidylcholine (OxPC) leading to cell death and pretreatment of cardiomyocytes with alpha linolenic acid (ALA) can reduce OxPC levels and improve cell survival during I/R [25].

Considering that PLs constitute 50% of total kidney lipid mass [26], their changes during I/R may contribute to I/R injury. Given that fragmented OxPC are potent apoptotic molecules, the aim of this study was to identify and measure these compounds during renal I/R injury.

## Material and methods

### Chemicals

HPLC grade solvents were obtained from VWR International (Mississauga, Ontario, Canada). The standard phospholipids; 1,2-dinonanoyl-sn-glycero-3-phosphocholine (09:0 PC), 1-heptadecanoyl-2-hydroxy-sn-glycero-3-phosphocholine (17:0 LPC), 1-palmitoyl-2-linoleoyl-sn-glycero-3-phosphocholine (PLPC), 1-palmitoyl-2-arachidonoyl-sn-glycero-3-phosphocholine (PAPC), 1-stearoyl-2-linoleoyl-sn-glycero-3-phosphocholine (SLPC), 1-stearoyl-2-arachidonoyl-sn-glycero-3-phosphocholine (SAPC), 1-palmitoyl-2-docosahexaenoyl-sn-glycero-3-phosphocholine (PDHPC), 1-palmitoyl-2-(5'-oxo-valeroyl)-sn-glycero-3-phosphocholine (POVPC), 1-palmitoyl-2-azelaoyl-sn-glycero-3-phosphocholine (PAzPC) and 1-palmitoyl-2-(9'-oxo-nonanoyl)-sn-glycero-3-phosphocholine (PONPC) were obtained from Avanti Polar Lipids (Alabaster, AL, USA). 1-palmitoyl-2-glutaryl-sn-glycero-3-phosphocholine (PGPC), 1-palmitoyl-2-(5’-keto-6’-octenedioyl)-sn-glycero-3-phosphocholine (KOdiA-PC) and 1-palmitoyl-2(4’-keto-dodec-3’-ene-dioyl)-sn-glycero-3-phosphocholine (KDdiA-PC) were purchased from Cayman Chemicals (Ann Arbor, Michigan, USA). All other chemicals were purchased from Sigma Aldrich (St. Louis, Missouri, USA).

### Renal ischemia–reperfusion (I/R)

Kidney ischemia was induced in 8 male Sprague–Dawley rats (250–300 g) by clamping the left renal pedicle for 45 min followed by reperfusion for 24h (n = 4 in each group). In brief, rats were anesthetized by 3% isoflurane/oxygen gas prior to surgery. Surgery was performed when rats reached stage 3 anesthesia. During surgery, 1–2% isoflurane/oxygen gas was maintained via inhalation. Rats were kept on a heat pad and the rectal temperature was maintained at 37°C throughout the experimental procedure. To prevent decreases in body temperature, rats were placed in a warm incubator for 12h after surgery. As a control, a sham‐operated group of rats were subjected to the same surgical procedure, but without inducing I/R, and were euthanized at corresponding time points. A blood sample was collected and plasma was separated by centrifugation at 3,000 g for 20 min at 4°C. A portion of kidney tissue was used for lipid extraction and the other portions used for staining. Kidney sections were stained with a hematoxylin and eosin (HE) staining kit (Baibo Biotechnology Co., Ltd., Shandong, China).

All procedures were performed in accordance with the Guide to the Care and Use of Experimental Animals published by the Canadian Council on Animal Care and approved by the University of Manitoba Protocol Management and Review Committee (permit number: B2015-039). Plasma creatinine concentrations were measured using a commercial assay kit (Genzyme Diagnostics, Canada). Harvested kidneys were rinsed in ice‐cold potassium phosphate buffer and then were snap frozen in liquid nitrogen and kept in -80 freezer to be used for analysis. Then, at the time of lipid extraction, they were pulverized in liquid nitrogen. Creatinine levels in plasma were measured using the Cobas C111 Analyzer (Roche, Laval, QC, Canada).

### Phospholipid extraction from renal tissue

Frozen renal tissue was thawed on ice. Rat kidney was pulverized in liquid nitrogen until a fine powder was obtained. A portion of each sample (30–50 mg) was weighed and subjected to lipid extraction with 2:1 (vol/vol) chloroform:methanol using the method described by Folch *et al* [27]. An internal standard mixture of 09:0 PC (0.1 μg/ml), 20:0 PC (1 μg/ml), 17:0 PC (LPC) (0.1 μg/ml), 17:0 PE (1 μg/ml), 16:0 PS (1 μg/ml), 14:0 CL (2 μg/ml), 14:0 PG (1 μg/ml), and 17:0–14:1 PI (1 μg/ml) was added to each sample prior to lipid extraction. A portion of lipid extract was reconstituted in mobile phase prior to injection on the LC/MS/MS system.

### Reverse-phase HPLC

Separation of OxPC species was done as described previously [25]. Briefly, 30 μL of sample extracts, reconstituted in reverse phase solvent, were injected onto an Ascentis Express C18 HPLC column (15 cm × 2.1 mm, 2.7 μm; Supelco Analytical, Bellefonte, Pennsylvania, USA) using a Prominence HPLC system (Shimadzu Corporation, Canby, Oregon, USA). Elution was performed by linear gradient of solvent A (acetonitrile/water, 60:40 vol/vol) and solvent B (isopropanol/acetonitrile, 90:10, vol/vol) both solvents containing 10 mM ammonium formate and 0.1% formic acid. The time program used was as follows: initial solvent B at 32%, increased to 45% B until 4.00 min; 5.00 min 52% B; 8.00 min 58% B; 11.00 min 66% B; 14.00 min 70% B; 18.00 min 75% B; 21.00 min 97% B; 25.00 min 97% B; 25.10 min 32% B until the elution was stopped at 30.10 min. A flow rate of 0.26 ml/min was used for analysis. The temperature of the column oven and sample tray was maintained at 45 and 4°C, respectively.

### Normal-phase HPLC

For the separation of non-oxidized phospholipids, 10 μL of sample extract reconstituted in normal phase solvent was injected onto an Ascentis Si HPLC column (15 cm × 1mm, 3 μm; Supelco Analytical, Bellefonte, Pennsylvania, USA) [28]. Elution was performed by linear gradient of solvent A (chloroform/methanol/ammonium hydroxide, 80:19.5:0.5 vol/vol/vol) and solvent B (chloroform/methanol/water/ammonium hydroxide, 60:34:5.5:0.5 vol/vol/vol/vol). The normal phase time program used was initial solvent B at 0%; ramped to 100% B at 14.00 min; held until 24.00 min at 100% B; then dropped at 24.10 min to 0% B until the elution was stopped at 30.10 min. A flow rate of 0.070 ml/min was used for analysis. The temperature of the column oven and sample tray was maintained at 25 and 4°C, respectively.

### Mass spectrometry

The HPLC system was coupled to a 4000 QTRAP® triple quadrupole linear ion trap hybrid mass spectrometer system equipped with a Turbo V electrospray ion source (AB Sciex, Framingham, Massachusetts, USA) [16]. OxPC, PC, LPC (lyso phosphatidylcholine) and SM (sphingomyelin) species were detected in positive ion mode via Multiple Reaction Monitoring (MRM) using the product ion 184.3 *m/z*, Da corresponding to the fragmented phosphatidylcholine head group. PI (Phosphatidylinositol), PG (phosphatidylglycerol) and PE (phosphatidylethanolamine) were detected in negative ion mode via MRM using the product ions 241, 170, and 139.8 *m/z*,Da, respectively. Product ions for PS are presented in supplementary materials (S1 Table). The mass spectrometer settings for positive ion mode analyses were as follows: electrospray ionization (ESI), 5500 V; declustering potential (DP), 125 V; entrance potential (EP), 10 V; collision energy (CE), 53 V; collision cell exit potential (CXP), 9V; curtain plate (CP), 26 psi; ion source gas 1 (GS1), 40 psi; ion source gas 2 (GS2), 30 psi, a retention time window of 50 msec was used and the temperature of the ion source was 500°C. The mass spectrometer settings for negative ion mode analyses were: ESI, -4500 V; DP, -80 V; EP, -10 V; CE, -60 V; CXP, -20V; CP, 20 psi; GS1, 30 psi; GS2, 30 psi, a retention time window of 100 msec and the temperature of the ion source was 500°C. Data was collected utilizing Analyst® Software 1.6 (AB Sciex). MultiQuant® Software 2.1 (AB Sciex) was used to compare peak areas of specific phospholipid compounds with their respective internal standard. Relative amounts of each phospholipid or oxidized phospholipid were then calculated based upon the amount of internal standard added. Final results are presented as the amount of phospholipid detected per mg of renal tissue extracted.

## Statistical analysis

One-way analysis of variance (ANOVA) with a Tukey post-hoc test for multiple comparisons was used to determine statistically significant differences between the three groups using a *P*-value < 0.05 (SPSS Software version 24, IBM Corporation, Armonk, NY, USA). Pearson’s correlation analysis was used to determine significant associations between study variables. All data are presented as mean ± SEM.

## Results

### Creatinine concentrations, histological analysis, and I/R injury

**Fig 1:**
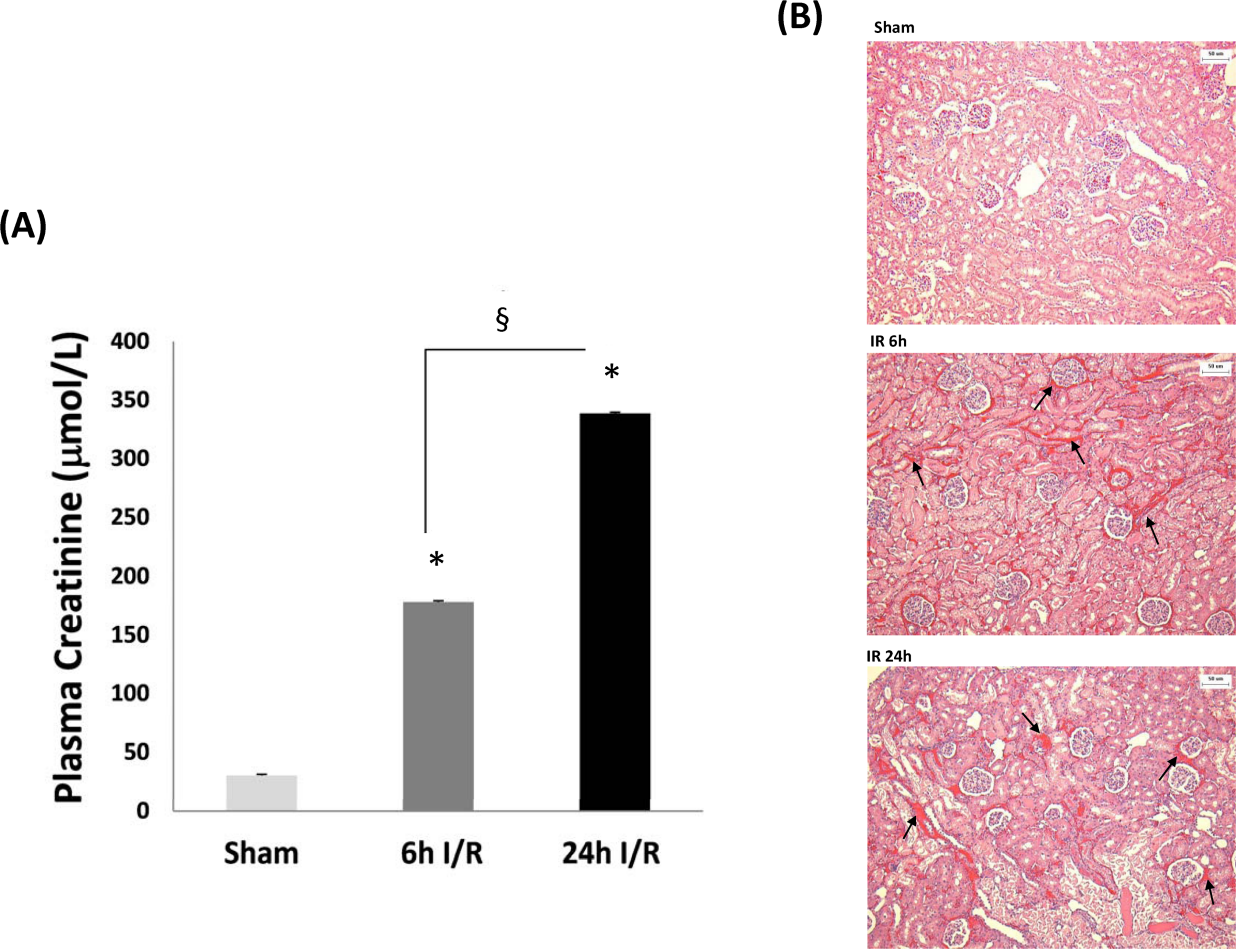
**Markers of acute kidney injury measured in (A) plasma and (B) renal slices from sham, 6 and 24h I/R groups.** (A) Plasma creatinine concentrations from sham, 6 and 24 hour I/R groups. Values are means ± SEM (n = 4 in each group). * Significant difference compared to sham, P<0.05. § Significant difference between I/R groups, P<0.05. (B) Hematoxylin and eosin (H&E) staining of renal tissue in sham, 6 and 24h I/R groups. The left kidney of rats was subjected to 45 min ischemia followed by 6h or 24h of reperfusion (I/R). As a control, rats were subjected to a sham-operation without inducing ischemia (Sham). The gross appearance of a mid-transverse plane of kidney was examined by H&E staining and analyzed at ×100 magnification. Kidneys in the I/R group followed by 6h or 24h of reperfusion (b and c) showed interstitial congestion (arrow) compared with the Sham group (a).

Plasma creatinine concentration, a marker of acute kidney injury, was assessed in each of the three experimental groups. Levels in plasma significantly increased both 6 and 24h after reperfusion (*P*<0.05) relative to the sham group (Fig 1A). Creatinine concentrations were statistically greater at 24h I/R compared to 6h I/R (*P* = 0.001). Hematoxylin and eosin (H&E) staining revealed that kidneys in the I/R groups showed interstitial congestion compared with the sham group (Fig 1B).

### OxPC in rat renal tissue following I/R injury

**Fig 2:**
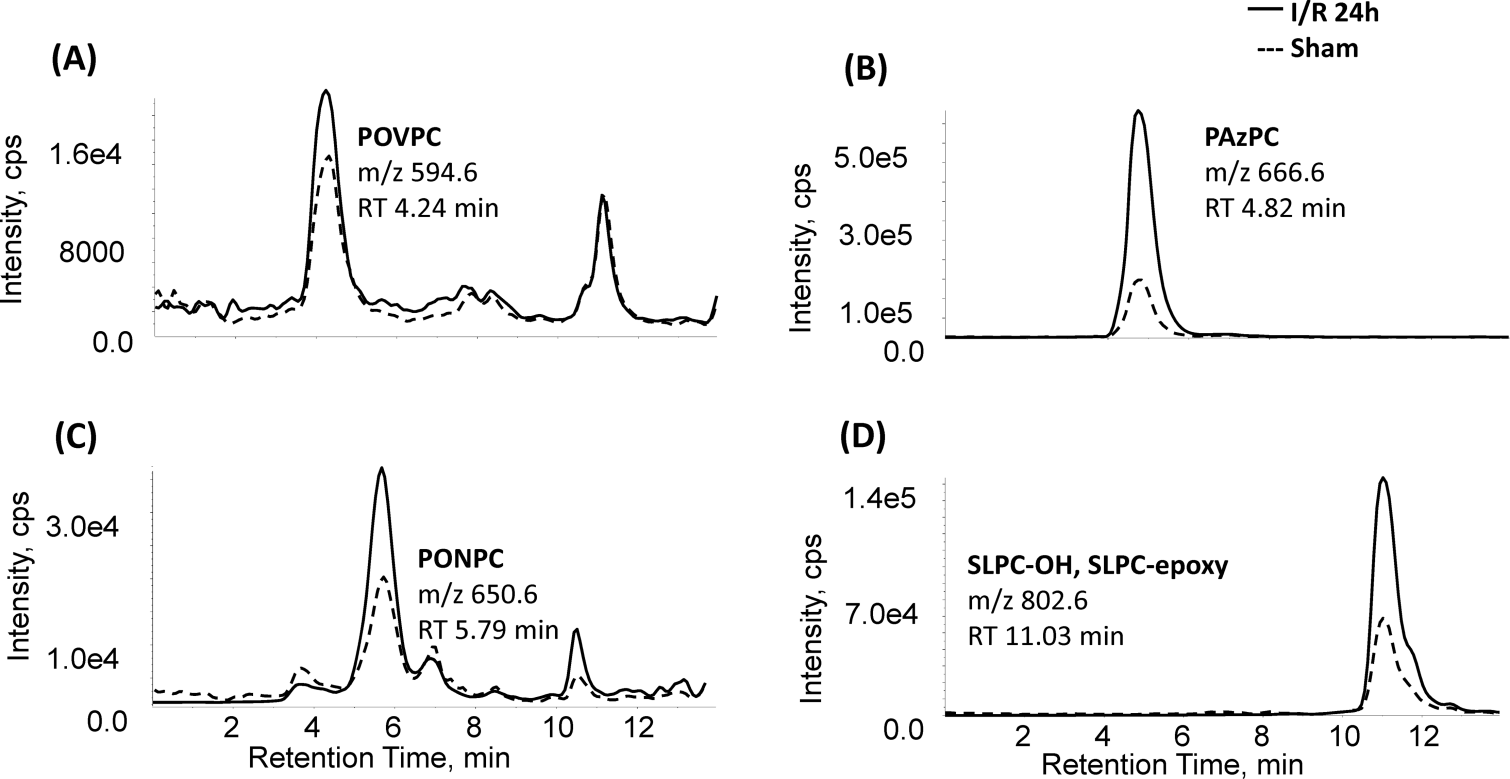
Fragmented and non-fragmented OxPC identified in rat renal tissue in sham operated and 24h I/R groups by LC/MS/MS. MRM chromatogram of (A) POVPC, (B) PAzPC, (C) PONPC and (D) SLPC-OH in renal tissue in I/R 24h (black line) and sham (dotted line) groups as measured by reverse phase HPLC-MS/MS. Abbreviations: POVPC, 1-palmitoyl-2-(5'-oxo-valeroyl)-sn-glycero-3-phosphocholine; PAzPC, 1-palmityl-2-azelayl-sn-glycero-3-phosphocholine; PONPC, 1-palmitoyl-2-(9'-oxo-nonanoyl)-sn-glycero-3-phosphocholine; SLPC-OH, 1-stearoyl-2-linoleoyl-phosphatidylcholine.

Mass spectral analysis of kidney lipid extracts identified fifty-five distinct OxPC in rat kidney following I/R injury. These included a variety of fragmented (aldehyde and carboxylic acid containing species) and non-fragmented (terminal furans, isoprostanes and long-chain products) OxPC derivatives. Representative chromatograms of four prominent OxPC species detected in renal tissue from sham (dashed line) and 24h I/R (solid line) are presented in Fig 2. These include the fragmented species POVPC (A), PAzPC (B), and PONPC (C) as well as the non-fragmented OxPC, SLPC-OH. For each OxPC identified, there was a significant increase in relative intensities in the I/R group relative to sham.

**Fig 3:**
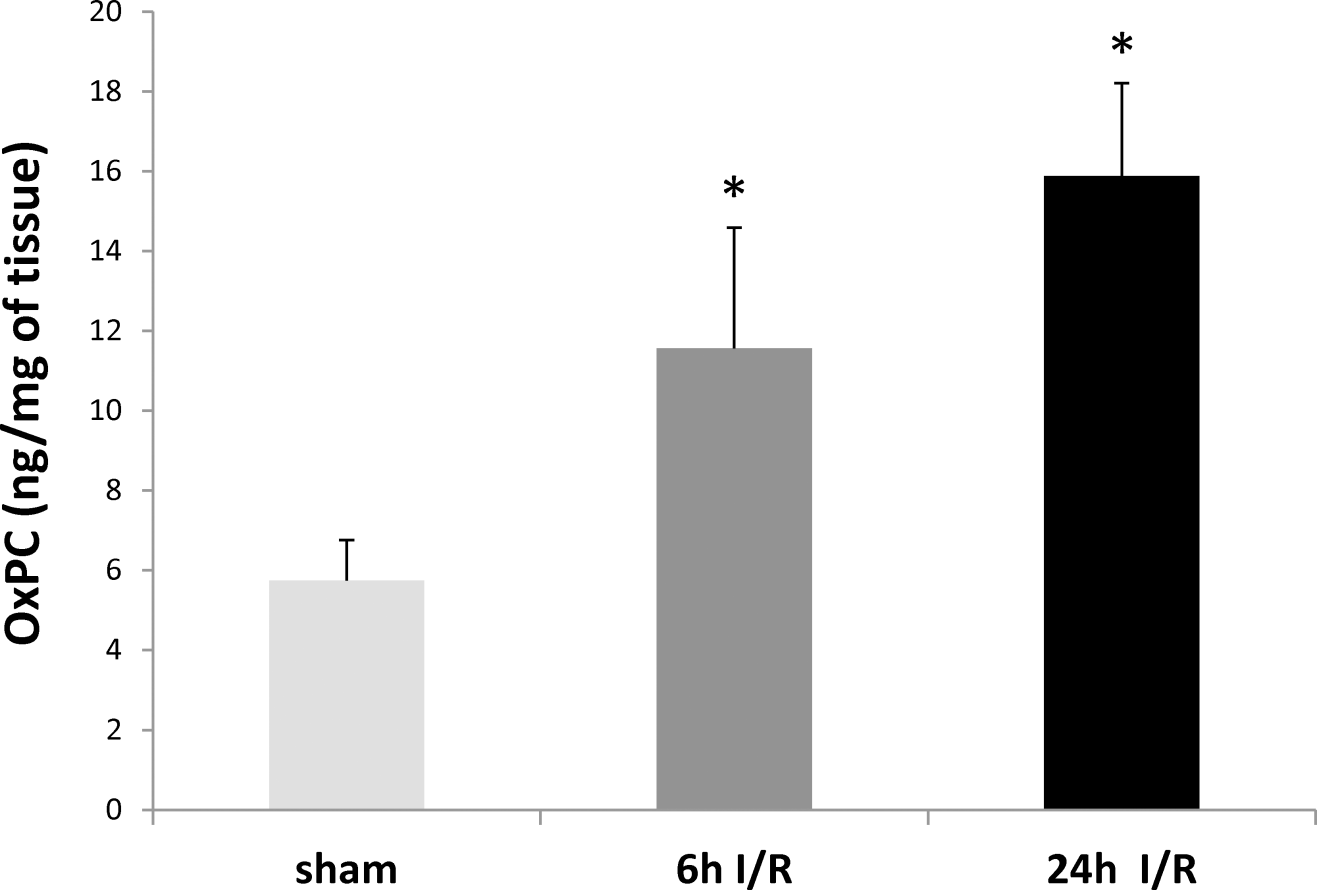
Total OxPC levels during renal I/R injury including fragmented and non-fragmented OxPC species measured in renal tissue in sham (light gray bar), 6h I/R (dark gray bar) and 24h I/R (black bar) groups. Values are means ± SEM (n = 4 in each group). * Significant difference compared to sham, *P*<0.05. Abbreviation: OxPC: Oxidized phosphatidylcholine.

Total OxPC levels significantly increased following both 6h (*P* = 0.01) and 24h (*P* = 0.001) reperfusion when compared to the sham group. There was a trend towards increase in total OxPC levels at 24h I/R compared to 6h I/R, but it did not reach statistical significance (*P* = 0.06) (Fig 3).

**Fig 4:**
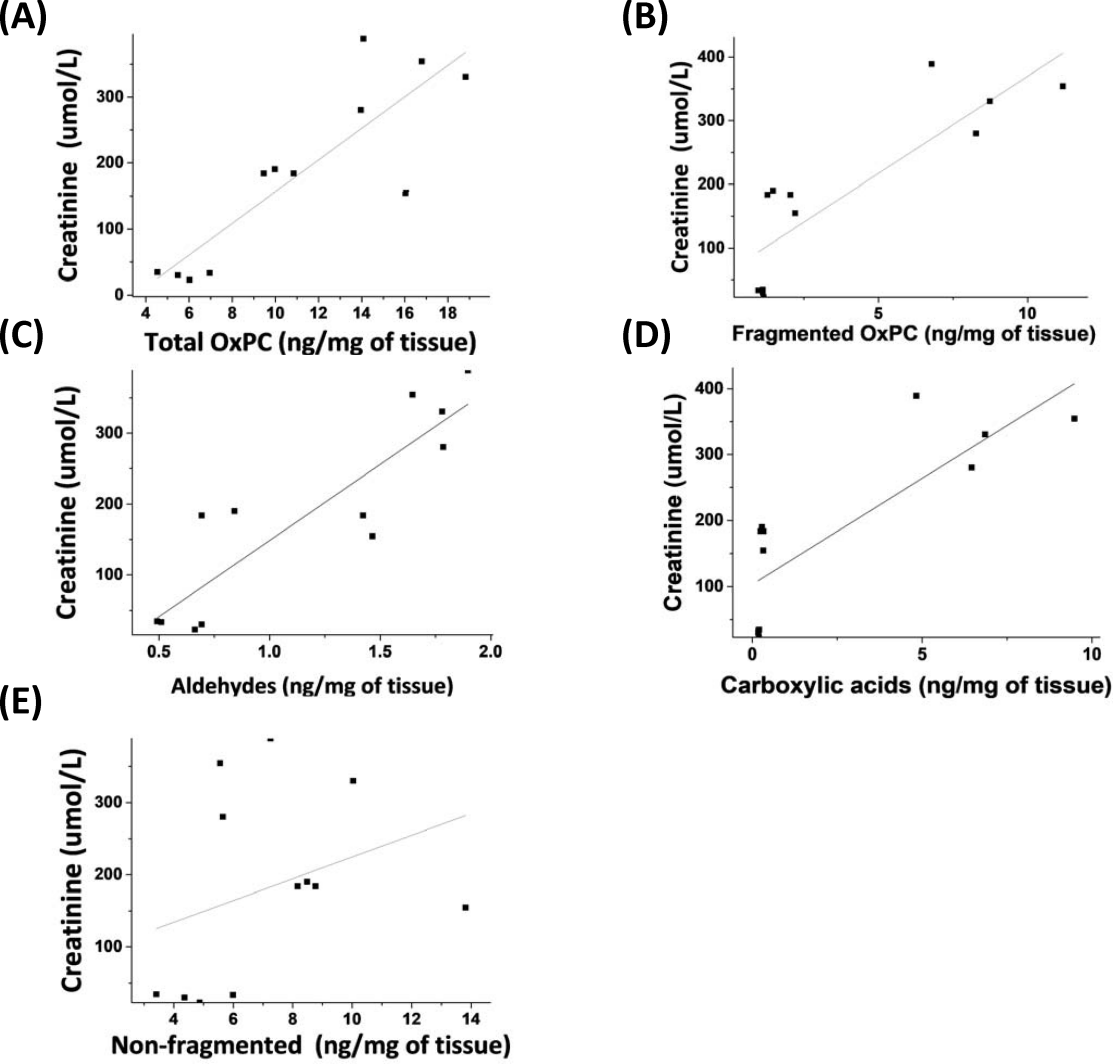
Correlations between OxPC species and creatinine levels. Correlations between (A) total OxPC (r = 0.863, *P* = 0.001), (B) fragmented OxPC (r = 0.885, *P* = 0.001), (C) aldehyde (r = 0.888, *P* = 0.001) and (D) carboxylic acid containing OxPCs (r = 0.821, *P* = 0.001), non-fragmented OxPC (r = 0.324, *P* = 0.305) and creatinine levels. n = 4 in each group. Abbreviation: OxPC: Oxidized phosphatidylcholine.

A significant positive correlation was observed between total OxPC levels and severity of AKI as measured by creatinine levels (r = 0.863, *P* = 0.001) (Fig 4A). Similar associations were observed for the OxPC subspecies, total fragmented (4B), aldehydes (4C) and carboxylic acids (4D), with the exception of total non-fragmented OxPCs (4E).

**Fig 5:**
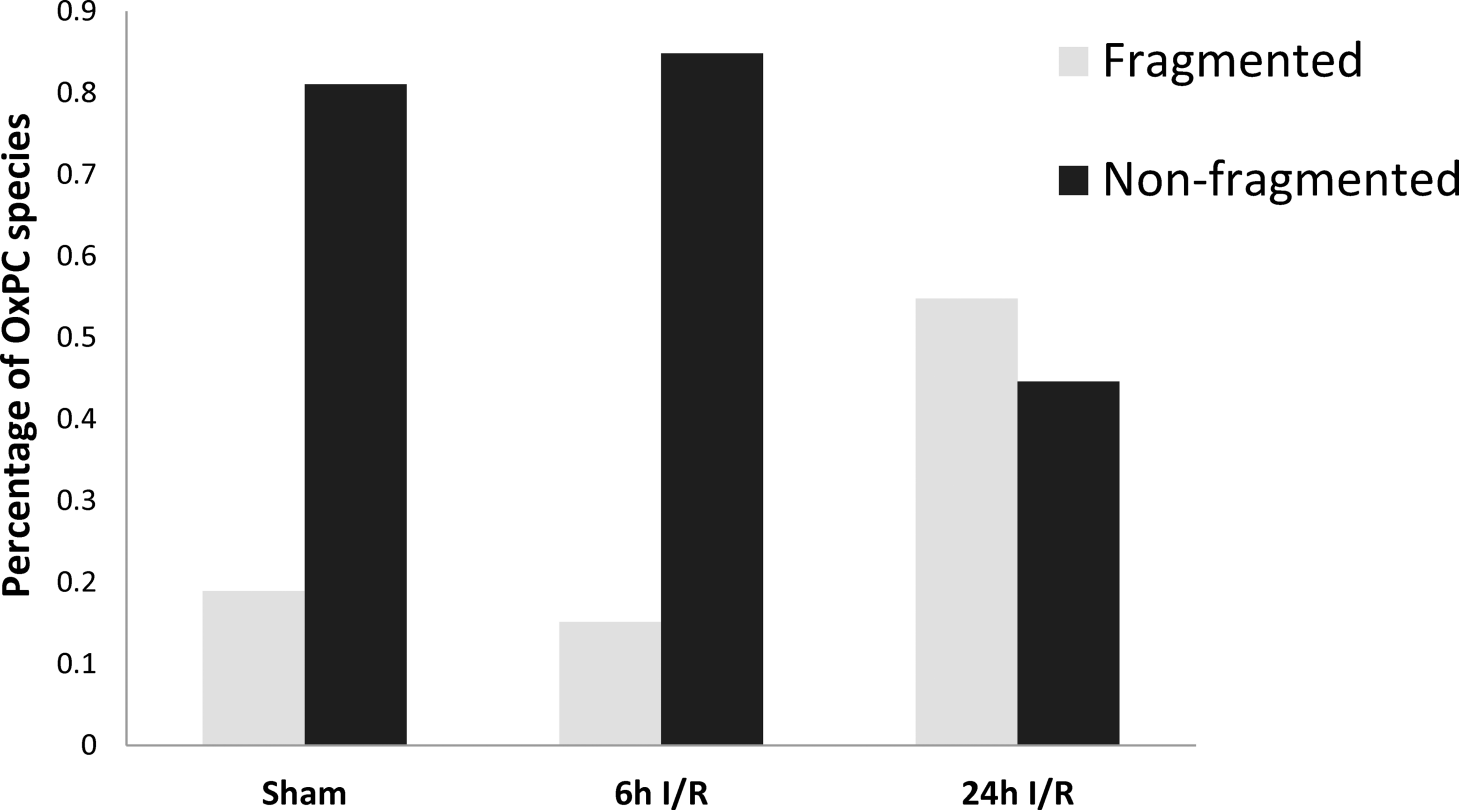
Percent of total OxPC in rat kidney in sham and I/R groups. Values represent the average relative percentage of a particular OxPC species compared to the total OxPC. n = 4 in each group, OxPC: Oxidized phosphatidylcholine.

To investigate which groups of OxPCs contributed most to the overall change in OxPC levels in each of the sham, 6h and 24h study groups, compounds were subdivided into fragmented and non-fragmented species. Percentages of total fragmented and non-fragmented OxPC in all groups are shown in Fig 5.

**Fig 6:**
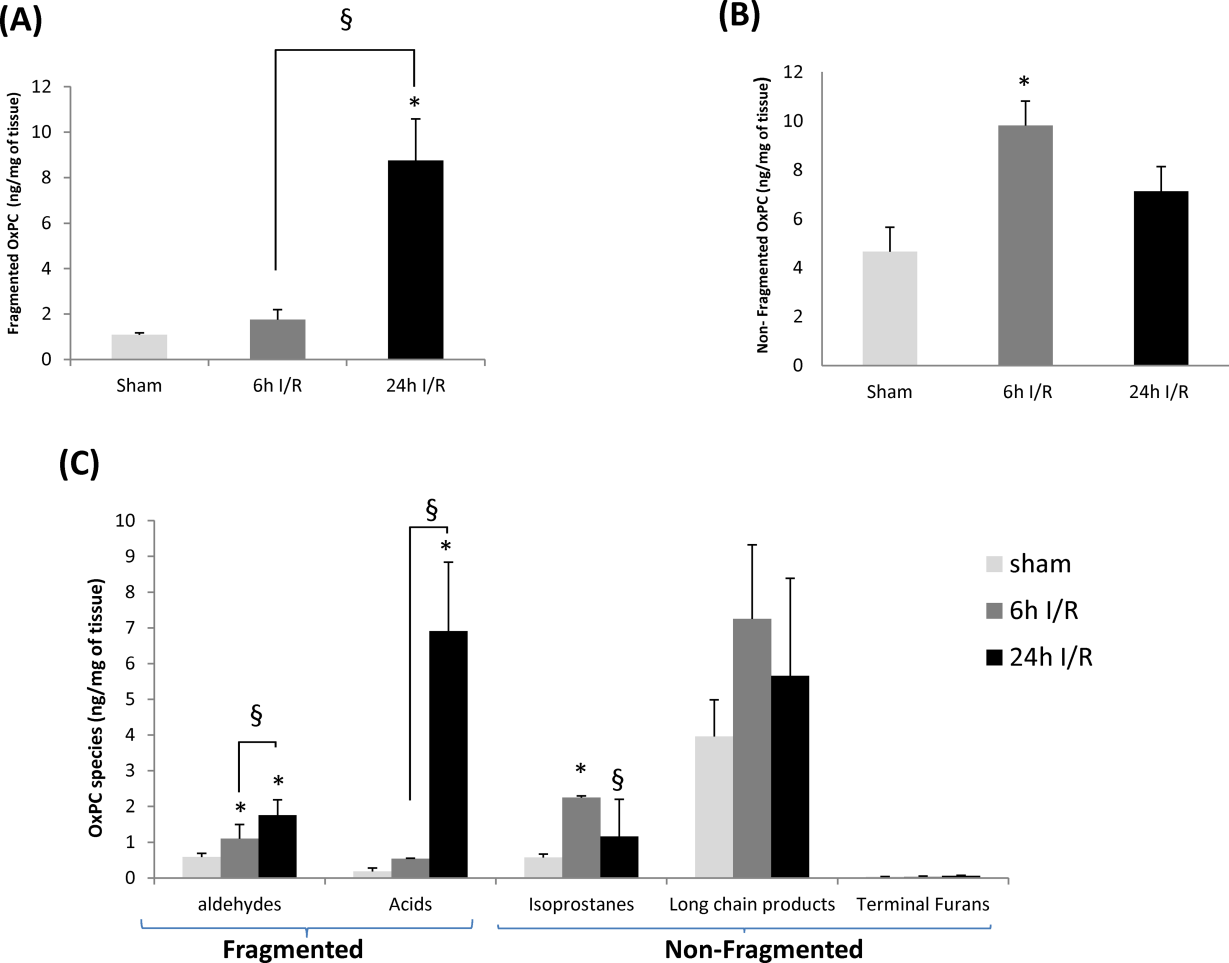
OxPC subgroups classified by fragmentation pattern and species in sham, 6h and 24h I/R groups. Total (A) fragmented OxPC, (B) non-fragmented OxPC and (C) OxPC by species in sham (light gray bar), 6h I/R (dark gray bar) and 24h I/R (black bar) groups. * Significant difference compared to sham, *P*<0.05. § Significant difference between I/R groups, *P*<0.05. Abbreviation: OxPC: Oxidized phosphatidylcholine.

Total fragmented OxPC increased significantly in the 24h I/R experimental group when compared to both sham-operated and 6h I/R groups (8.74±1.83 versus 1.08±0.07 and1.75±0.44 ng/mg of renal tissue, *P* = 0.001) (Fig 6A). However, no significant differences were observed between sham and 6h I/R groups. When correlated with creatinine levels to assess AKI severity, a strong positive correlation was observed between total fragmented OxPC levels and creatinine levels (r = 0.885, *P* = 0.001) (Fig 4B).

The most abundant fragmented OxPC measured in renal tissue after 6 and 24h I/R were PONPC and PAzPC. Average amounts consisted of 0.25±0.09 and 0.24±0.03 ng/mg after 6h and 0.57±0.02 and 6.37±1.99 ng/mg of renal tissue after 24h, respectively (Table 1). All fragmented OxPC species identified in rat kidney tissue are presented in Table 1.

**Table 1:** Identified fragmented OxPC species in kidney tissue in sham and I/R groups.

Fragmented OxPC	Sham (ng/mg of tissue)	6h I/R (ng/mg of tissue)	24h I/R (ng/mg of tissue)
**4-oxo-butyryl-PC**	0.04±0.00	0.06±0.02	0.09±0.00*^§^
**POVPC**	0.1±0.02	0.18±0.07	0.27±0.01*
**Succinoyl-PC**	0.03±0.00	0.07±0.01*	0.08±0*
**PGPC**	0.02±0.01	0.03±0.01	0.07±0.01*
**SOVPC**	0.17±0.04	0.31±0.11	0.36±0.06*
**Furylbutanoyl-PC**	0.01±0.00	0.01±0.00	0.02±0.00
**KOHA-PC**	0.01±0.00	0.01±0.00	ND
**8-oxo-octanoyl-PC**	0.04±0.00	0.07±0.03	0.11±0.01*
**SGPC**	0.06±0.00	0.06±0.04	0.07±0.02
**KOOA-PC**	ND	0.01±0.01	0.04±0.01*
**PONPC**	0.13±0.02	0.25±0.09*	0.57±0.02*
**Furylbutanoyl-SPC**	0.01±0.00	0.02±0.01	0.03±0.02
**KOdiA-PC**	0.04±0.01	0.07±0.03	0.10±0.02*
**PAzPC**	0.21±0.08	0.24±0.03	6.37±1.99*^§^
**KOOA-SPC**	ND	ND	0.01±0.00*
**SONPC**	0.06±0.01	0.16±0.06*	0.28±0.02*
**Furyloctanoyl-PC**	0.01±0.00	0.00±0.00	0.01±0.00
**SAzPC**	0.08±0.02	0.10±0.01	0.29±0.13*
**HODA-PC**	0.002±0.00	0.003±0.00	0.007±0.00*^§^
**HDdiA-PC**	ND	ND	0.01±0.00*

Abbreviations: POVPC: 1-palmitoyl-2-(5'-oxo-valeroyl)-sn-glycero-3-phosphocholine, PGPC: 1-palmitoyl-2-glutaryl-sn-glycero-3-phosphocholine, SOVPC: sn-1-stearoyl-2-oxovaleroyl-PC, KOHA-PC: 1-palmityl-2-(4-oxo-7-oxohept-5-enoyl)-sn-glycero-3-phosphoserine, SGPC: 1-stearoyl-2-glutaroyl-sn-glycero-3-phosphocholine, KOOA-PC: 5-hydroxy-8-oxo-6-octenoic acid ester of 2-lysophosphatidylcholine, PONPC: 1-palmitoyl-2-(9'-oxo-nonanoyl)-sn-glycero-3-phosphocholine, KOdiA-PC: 1-palmitoyl-2-(5’-keto-6’-octenedioyl)-sn-glycero-3-phosphocholine, PAzPC: 1-palmityl-2-azelayl-sn-glycero-3-phosphocholine, **KOOA-SPC,** SONPC: 1-stearoyl -2-(9-oxo-nonanoyl)-snglycero-3-phosphocholine, SAzPC: 1-stearoyl-2-azelaoyl-sn-glycero-3-phosphocholine, HODA-PC: 9-hydroxy-12-oxododec-10-enoic acid ester of 2-lysophosphatidylcholine, HDdiA-PC: 1-palmitoyl-2-(9-hydroxy-11-carboxyundec-6-enoyl)-sn-glycero3-phosphocholine.

* Significant differences compared with sham group.

^§^ Significant differences compared with 6h I/R group, *P*<0.05.

Fragmented OxPC were then classified into two groups: aldehyde and carboxylic acid containing OxPC. Total aldehydes were significantly higher in the 6h and 24h I/R groups in comparison to the sham group. Moreover, levels of aldehyde OxPC at 24h I/R were significantly greater than those in 6h I/R (*P* = 0.007) (Fig 6B). Significant correlations were also observed between levels of aldehyde containing OxPC and plasma creatinine levels (r = 0.888, *P* = 0.001) (Fig 4C). For total fragmented carboxylic acids, significant increases were observed in the 24h I/R group compared with both sham and 6h I/R group (6.91±1.92 versus 0.18±0.09 and 0.53±0.01 ng/mg of renal tissue, *P* = 0.001) (Fig 6B). No significant changes were identified between sham-operated and 6h I/R group. Similar to aldehyde containing OxPC, levels of carboxylic acid containing OxPC were positively correlated with severity of I/R injury as measured by creatinine levels (r = 0.821, *P* = 0.001) (Fig 4D).

Non-fragmented species also had significant changes as a result of I/R injury (Fig 6C). The most abundant non-fragmented OxPC after 6h and 24h I/R was SLPC-OH (2.49±1.62 and 1.87±0.41 ng/mg of tissue) (Table 2). Total non-fragmented OxPC concentrations in the 6h I/R group were significantly greater than the sham group, but not at 24h I/R (Fig 6C). This is likely attributed to the high prevalence of isoprostanes after 6h I/R (Fig 6B). IsoPG (E2,I2,D2)-SPC, the most prevalent isoprostane identified in renal tissue after 6h I/R, was detected at concentrations of 0.46±0.19 ng/mg tissue with 0.071±0.02 ng/mg tissue in sham tissue. Although non-fragmented isoprostanes increased significantly in the 6h I/R group compared with sham group, their levels dropped significantly at 24h I/R when compared to the 6h group (*P* = 0.02) and were unchanged compared to control (*P* = 0.488) (sham: 0.57±0.10, 6h I/R: 2.24±0.04 and 24h I/R: 1.16±1.03 ng/mg of renal tissue). Non-fragmented OxPC derivatives including long-chain products and terminal furans were unchanged in any of the I/R groups (Fig 6B). Interestingly, no significant correlations were observed between any of the non-fragmented OxPC (including total non-fragmented OxPC, terminal furans, isoprostanes and long-chain products) with creatinine levels (r = 0.324, *P* = 0.305). All non-fragmented OxPC species identified are presented in Table 2.

**Table 2:** Identified non-fragmented OxPC species in kidney tissue in sham and I/R groups.

Non Fragmented OxPC	Sham (ng/mg of tissue)	6h I/R (ng/mg of tissue)	24h I/R (ng/mg of tissue)
**PLPC-keto**	0.19±0.04	0.4±0.14	0.53±0.246*
**PLPC-OH**	0.19±0.05	0.32±0.10	0.27±0.158
**PLPC-epoxy,ketoPLPC-OH,keto**	0.12±0.04	0.31±0.15	0.2±0.053
**PLPC-OOH**	0.09±0.03	0.18±0.07	0.14±0.101
**PAPC-keto**	0.16±0.05	0.42±0.20	0.36±0.185
**PAPC-OH**	0.09±0.02	0.28±0.22	0.17±0.093
**SLPC-keto**	0.18±0.06	0.51±0.21*	0.35±0.134
**SLPC-OH**	1.73±0.56	2.49±1.62	1.87±0.417
**PLPC-OOH,keto**	0.01±0.00	0.01±0.01	0.01±0.011
**PLPC-OOH,OH**	0.11±0.08	0.12±0.05	0.14±0.07
**PLPC-diOH,epoxy**	0.06±0.10	0.05±0.06	0.02±0.00
**isoPG(A2,J2)-PC**	0.05±0.02	0.17±0.05*	0.08±0.05
**PAPC-OOH**	0.04±0.03	0.06±0.06	0.02±0.01
**SLPC-epoxy,keto**	0.10±0.03	0.25±0.07*	0.24±0.08
**SLPC-OOH**	0.05±0.03	0.09±0.03	0.06±0.04
**PGJ2-SPC**	0.06±0.01	0.17±0.05	0.29±0.10*
**SAPC-keto**	0.10±0.03	0.30±0.06*	0.35±0.13
**SAPC-OH**	0.53±0.24	0.73±0.46	0.43±0.11
**PEIPC**	0.03±0.02	0.10±0.06	0.06±0.02
**isoPG(E2,I2,D2)-PC**	0.09±0.05	0.16±0.10	0.08±0.05
**isoPGF2α-PC**	0.04±0.02	0.18±0.03*	0.09±0.04
**SAPC-OH**	0.53±0.24	0.73±0.46	0.43±0.11
**SLPC-OOH**	0.05±0.03	0.09±0.03	0.06±0.04
**SLPC-triOH**	0.04±0.01	0.08±0.02*	0.05±0.01
**SECPC**	0.05±0.00	0.23±0.05*	0.12±0.04
**isoPG(A2,J2)-SPC**	0.03±0.00	0.17±0.05*	0.10±0.06
**SAPC-OOH**	0.05±0.01	0.38±0.15*	0.26±0.13
**SEIPC**	0.07±0.01	0.27±0.02*	0.11±0.04
**isoPG(E2,I2,D2)-SPC**	0.07±0.02	0.46±0.19*	0.18±0.12
**isoPGF2α-SPC**	0.02±0.00	0.15±0.07*	0.05±0.02
**SLPC-OOH,OH**	0.01±0.00	0.05±0.03	0.04±0.03
**SLPC-triOH**	0.05±0.00	0.23±0.05	0.12±0.04
**SECPC**	0.03±0.00	0.17±0.05	0.12±0.06
**isoPG(A2,J2)-SPC**	0.05±0.01	0.38±0.15	0.26±0.13
**SAPC-OOH**	0.07±0.01	0.27±0.02	0.11±0.04
**SEIPC**	0.07±0.02	0.46±0.19	0.18±0.12
**isoPG(E2,I2,D2)-SPC**	0.02±0.00	0.15±0.07	0.05±0.02
**isoPGF2α-SPC**	0.05±0.00	0.23±0.05	0.12±0.04
**SAPC-OOH,diketo**	0.02±0.00	0.04±0.00*	0.02±0.01
**SAPC-OOH,OH,keto**	0.04±0.01	0.08±0.02*	0.05±0.02
**SAPC-diOOH**	0.05±0.00	0.17±0.01*	0.08±0.04
**Isofuran-SPC**	0.01±0.00	0.12±0.08*	0.05±0.03
**SAPC-OOH,OH,epoxy**	0.00±0.00	0.00±0.00*	0.01±0.00
**SAPC-diOOH,OH**	0.02±0.00	0.07±0.03	0.04±0.02
**SAPC-triOOH**	0.02±0.00	0.03±0.00	0.03±0.01

PLPC: 1-palmitoyl-2-linoleoyl-sn-glycero-3-phosphocholine, PAPC: 1-palmitoyl-2-arachidonoyl-snglycero-3-phosphocholine, SLPC: 1-stearoyl-2-linoleoyl-sn-glycero-3- phosphocholine, isoPG: Isoprostanes, PGJ2-SPC: prostaglandin J2- stearoyl phosphocholine, SAPC: 1-stearoyl-2-arachidonoyl-sn-glycero-3-phosphocholine,PEIPC: 1-palmitoyl-2-(5,6-epoxyisoprostane E2)-sn-glycero-3-phosphocholine, SEIPC: 1- stearoyl -2-(5,6-epoxyisoprostane E2)-sn-glycero-3-phosphocholine,. OH: hydroxyl, OOH: hydroperoxy.

* Significant differences compared with sham group, P<0.05.

### Non-oxidized phospholipids in rat renal tissue following I/R injury

PC and sphingomyelin (SM) were the most abundant PLs in sham group (25.78±1.94 and 7.27±0.69 μg/mg of tissue, respectively), followed by PI (0.55±0.12 μg/mg of tissue) and PE (0.87±0.20 μg/mg of tissue), then PS (0.43±0.08 μg/ mg of tissue) and PG (0.025±0.003 μg/mg of tissue).

**Fig 7:**
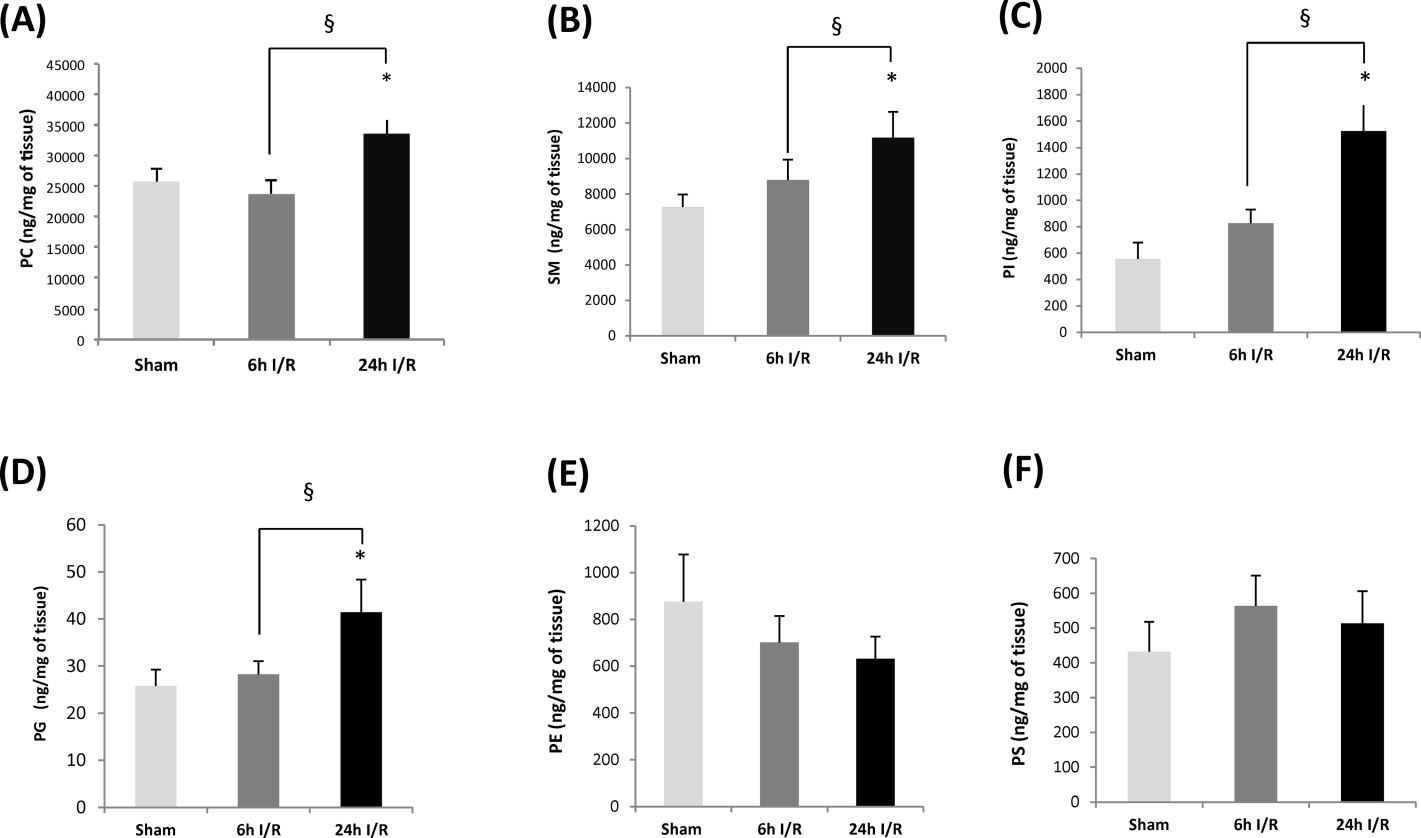
Representative amounts of non-oxidized lipids in renal tissue from sham, 6h and 24h I/R groups. Average (A) PC, (B) SM, (C) PI, (D) PG, (E) PE and (F) PS concentrations in rat renal tissue in sham and 6 and 24h I/R groups (n = 4 in each group). * Significant difference compared to sham, P< 0.05. § Significant difference between I/R groups, *P*<0.05. Abbreviations: PC: Phosphatidylcholine, SM: Sphingomyelin, PI: Phosphatidylinositol, PG: Phosphatidylglycerol, PE: Phosphatidylethanolamine, PS: Phosphatidylserine.

Average concentrations of total PC content in renal tissue for each of the experimental groups are represented in Fig 7A. Although there were no changes in total PC levels after 6h I/R compared to sham, PC levels significantly increased after 24h I/R when compared to both sham and 6h I/R groups (3.36±4.80 versus 2.57±1.94 and 2.37±2.41 μg/mg of renal tissue respectively *P* = 0.015, 0.004) (Fig 7A).

Thirty-six PC species were identified in renal tissue in all three experimental groups. Of these, PC (36:4) was the most abundant (Table 3). There were no significant increases in any PC after only 6h I/R; however, after 24h I/R, of the 36 individual PC compounds identified, 29 and 27 were significantly greater than those in the sham and 6h I/R groups, respectively. Of these, the top 10 PC compounds after 24h I/R, beginning with the most abundant, were PC(34:1) > PC(34:2) > PC(38:4) > PC(36:2) > PC(38:5) > PC(36:1) > PC(34:0) > PC(38:3) > PC(32:1) > PC(40:6) (Table 3). Of interest, three PC compounds were significantly reduced after 6h I/R compared to the sham group, including PC(34:2), PC(34:3) and PC(36:5). All identified PC species in kidney tissue are presented in Table 3.

**Table 3:** Identified PC species in kidney tissue in sham and I/R groups.

	Sham (μg/mg of tissue)	6h I/R (μg/mg of tissue)	24h I/R (μg/mg of tissue)
**PC(28:0)**	0.006±0.00	0.007±0.00	0.01±0.00
**PC(30:0)**	0.24±0.05	0.25±0.06	0.42±0.11*^§^
**PC(32:0)**	3.23±0.24	2.98±0.32	3.51±0.33
**PC(34:0)**	0.47±0.06	0.56±0.04	0.93±0.14*^§^
**PC(36:0)**	0.08±0.00	0.12±0.01	0.18±0.05*^§^
**PC(30:1)**	0.01±0.00	0.01±0	0.02±0.00*^§^
**PC(32:1)**	0.40±0.05	0.34±0.04	0.66±0.03*^§^
**PC(32:2)**	0.04±0.00	0.05±0	0.09±0.01*^§^
**PC(34:1)**	2.92±0.23	2.79±0.43	4.42±0.71*^§^
**PC(34:1 p)**	0.14±0.01	0.14±0.02	0.22±0.02*^§^
**PC(34:2)**	3.68±0.32	2.88±0.37*	4.31±0.39*
**PC(34:2p)**	0.1±0.01	0.09±0.01	0.14±0.01*^§^
**PC(34:3)**	0.15±0.02	0.12±0.00*	0.25±0.01*^§^
**PC(36:1)**	0.48±0.05	0.56±0.09	0.95±0.24*^§^
**PC(36:1p)**	0.05±0.00	0.05±0.00	0.08±0.01*^§^
**PC(36:2)**	1.63±0.16	1.56±0.23	2.35±0.36*^§^
**PC(36:2p)**	0.06±0.00	0.05±0.01	0.09±0.01*^§^
**PC(36:3)**	1.20±0.15	0.99±0.10	1.47±0.16
**PC(36:4)**	4.52±0.33	3.79±0.45	4.72±0.80
**PC(36:4p)**	0.33±0.04	0.37±0.04	0.47±0.14
**PC(36:5)**	0.31±0.04	0.21±0.02*	0.24±0.03*
**PC(36:6)**	0.01±0.00	0.01±0.00	0.02±0.00
**PC(38:2)**	0.06±0.00	0.07±0.01	0.11±0.02*^§^
**PC(38:3p)**	0.005±0.00	0.005±0.00	0.009±0.00*^§^
**PC(38:3)**	0.44±0.04	0.5±0.03	0.65±0.125*
**PC(38:4)**	2.35±0.17	2.59±0.22	3.47±0.67*^§^
**PC(38:5p)**	0.01±0.00	0.02±0.00	0.03±0.01*^§^
**PC(38:5)**	0.89±0.08	0.75±0.08	1.16±0.11*^§^
**PC(38:6)**	1.23±0.15	0.99±0.12	1.32±0.19^§^
**PC(40:2)**	0.00±0.00	0.00±0.00	0.01±0.00*^§^
**PC(40:3)**	0.00±0.00	0.01±0.00	0.02±0.00*^§^
**PC(40:4)**	0.03±0.00	0.05±0.01	0.1±0.02*^§^
**PC(40:5)**	0.09±0.00	0.13±0.02	0.25±0.05*^§^
**PC(40:6)**	0.22±0.00	0.3±0.03	0.5±0.12*^§^
**PC(40:7)**	0.09±0.00	0.09±0.01	0.14±0.01*^§^
**PC(40:8)**	0.14±0.01	0.13±0.02	0.14±0.02

Abbreviation: PC: Phosphatidylcholine

* Significant differences compared with sham group.

§ Significant differences compared with 6h I/R group, P<0.05.

Thirteen LPC compounds were detected in all groups. LPC (18:0) was the most abundant LPC species in all groups with average values representing 116.07±15.69, 118.35 ±5.30 and 108.56±16.26 ng/mg of tissue for sham-operated, 6h and 24h I/R groups, respectively (Table 4). No significant changes were detected in total LPC levels amongst any of the study groups (data not shown). However, LPC (16:0p) dropped significantly in 24h I/R group compared with 6h I/R group. LPC (20:3) decreased significantly in 24h I/R group in compared with sham and 6h I/R groups (*P* = 0.014, 0.005 respectively). There were also significant reductions in LPC (22:6) levels in 24h I/R group when compared to 6h I/R group (*P* = 0.019). On the contrary, LPC (22:5) levels were elevated significantly in 6h I/R group compared to sham operated group (*P* = 0.012), but no change was observed following 24h I/R. All identified LPC species in kidney tissue are presented in Table 4.

**Table 4:** Identified LPC species in kidney tissue in sham and I/R groups.

	Sham (ng/mg of tissue)	6h I/R (ng/mg of tissue)	24h I/R (ng/mg of tissue)
**LPC (14:0)**	0.06±0.00	0.08±0.02	0.06±0.02
**LPC(16:0)**	94.73±8.7	92.64±7	78.29±14.6
**LPC(16:0 Alkenyl)**	0.32±0.10	0.42±0.17	0.24±0.07
**LPC(16:0p)**	1.19±0.12	1.23±0.19	0.91±0.15^§^
**LPC(16:1)**	1.01±0.12	0.94±0.20	0.85±0.08
**LPC(18:0)**	116.07±15.69	118.35±5.30	108.56±16.26
**LPC(18:1)**	11.26±0.83	12.26±2.54	9.75±1.43
**LPC(18:2)**	11.09±0.70	11.24±2.71	9.41±1.64
**LPC(18:3)**	0.22±0.05	0.18±0.01	0.15±0.02
**LPC(20:3)**	1.27±0.00	1.38±0.30	0.75±0.15*^§^
**LPC(22:5)**	0.45±0.00	0.73±0.12*	0.55±0.11
**LPC(22:6)**	1.92±0.18	2.53±0.43	1.60±0.46^§^

Abbreviation: LPC: Lyso phosphatidylcholine

* Significant differences compared with sham group.

^§^ Significant differences compared with 6h I/R group, *P*<0.05.

SM was the next most prevalent PL species identified in renal tissue of sham and I/R groups. Mean total concentrations of SM increased with increasing I/R time; however, levels in the 24h I/R group (11.18±0.14 μg/mg tissue) were significantly greater than those in the sham-operated (7.27± 0.69 μg/mg of tissue; *P* = 0.002) and 6h I/R groups (8.79± 1.15 μg/mg of tissue, *P* = 0.029) (Fig 7B). No significant differences were detected between sham and 6h I/R groups. Of the 40 SM compounds identified, SM (34:1), SM (42:1) and SM (42:2) (Table 5) were the three most abundant SM compounds in all groups. All identified SM species in kidney are presented in Table 5.

**Table 5:** Identified SM species in renal I/R.

	Sham (μg/mg of tissue)	6h I/R (μg/mg of tissue)	24h I/R (μg/mg of tissue)
**SM 31:0**	ND	ND	0.001±0.00^§^*
**SM 33:0**	0.002±0.00	0.002±0.00	0.002±0.00
**SM 34:0**	0.30±0.01	0.39±0.05	0.43±0.06*
**SM 35:0**	0.009±0.00	0.01±0.00*	0.005±0.00
**SM 36:0**	0.02±0.00	0.02±0.00	0.031±0.00
**SM 38:0**	0.03±0.02	0.01±0.00	0.014±0.00
**SM 40:0**	0.02±0.00	0.03±0.00	0.091±0.02*
**SM 44:0**	0.003±0.00	0.005±0.00	0.011±0.00*
**SM 32:2**	0±0.00	0±0.00	0.001±0.00
**SM 32:1**	0.01±0.00	0.01±0.00	0.01±0.00
**SM 32:2i**	0.001±0.00	0.001±0.00	0.001±0.00
**SM 31:2**	0.001±0.00	0.002±0.00	0.002±0.00
**SM 31:1**	0.02±0.00	0.03±0.00	0.03±0.00
**SM 34:2**	0.14±0.01	0.19±0.03	0.23±0.02*
**SM 34:1**	2.99±0.22	3.36±0.58	3.53±0.53
**SM 35:1**	0.04±0.00	0.05±0.00	0.06±0.01
**SM 36:3**	0±0.00	0±0.00	0.001±0.00
**SM 36:2**	0.02±0.00	0.03±0.00	0.04±0.01
**SM 36:1**	0.18±0.02	0.25±0.03	0.33±0.06*
**SM 37:1**	0.006±0.00	0.007±0.00	0.01±0.00*
**SM 38:3**	0±0.00	0±0.00	0.001±0.00
**SM 38:1**	0.14±0.05	0.15±0.01	0.22±0.04*
**SM 39:2**	0.001±0.00	0.001±0.00	0.001±0.00
**SM 39:1**	0.009±0.00	0.01±0.00	0.01±0.00§*
**SM 40:3**	0.02±0.03	0.01±0.00	0.01±0.003
**SM 40:2**	0.05±0.01	0.07±0.00	0.1±0.00*
**SM 40:1**	0.29±0.06	0.36±0.05	0.55±0.10*
**SM 41:3**	0.006±0.00	0.007±0.00	0.01±0.00*
**SM 41:2**	0.02±0.00	0.03±0.00	0.04±0.00*
**SM 41:1**	0.07±0.01	0.09±0.01	0.15±0.02*
**SM 42:4**	0.02±0.00	0.03±0.00	0.05±0.00*
**SM 42:3**	0.31±0.08	0.35±0.04	0.57±0.09§*
**SM 42:2**	1.10±0.23	1.42±0.11	1.95±0.21§*
**SM 42:1**	1.24±0.24	1.64±0.217	2.35±0.25§*
**SM 43:3**	0.002±0.00	0.00±0.00	0.004±0.00^§^*
**SM 43:2**	0.01±0.00	0.01±0.00	0.02±0.00§*
**SM 43:1**	0.02±0.00	0.03±0.00*	0.05±0.00§*
**SM 44:3**	0.006±0.00	0.00±0.00	0.01±0.00§*
**SM 44:2**	0.02±0.00	0.03±0.00*	0.05±0.00§*
**SM 44:1**	0.03±0.00	0.04±0.00*	0.08±0.01§*

Abbreviation: SM: Sphingomyelin

* Significant differences compared with sham group.

^§^ Significant differences compared with 6h I/R group, P<0.05.

Total concentrations of PI followed a similar pattern as that observed for SM with concentrations significantly elevated in the 24h I/R group (1.52±0.20 μg/mg of tissue) compared to sham (0.55±0.12 μg/mg of tissue *P* = 0.001) and 6h I/R groups (0.82±0.10 μg/mg of tissue, *P* = 0.001) (Fig 7C). Twenty PI compounds were also identified in renal tissue in all groups. Of these, only 9 species increased significantly following 6h I/R, but all 20 PI species increased significantly following 24h I/R compared with the sham-operated group (Table 6). The most abundant PI compound was PI (38:4) with average values representing 0.31±0.07, 0.38±0.95 and 0.68±0.12 μg/mg of tissue in sham, 6h and 24h IR groups, respectively (Table 6).

**Table 6:** Identified PI species in renal I/R.

	Sham (μg/mg of tissue)	6h I/R (μng/mg of tissue)	24h I/R (μng/mg of tissue)
**PI 30:0**	ND	ND	0.003±0.00^§^*
**PI 32:0**	ND	ND	0.05±0.00^§^*
**PI 32:1**	0.00±0.00	0.00±0.00	0.006±0.00*
**PI 34:0**	ND	ND	0.05±0.00^§^*
**PI 34:1**	0.02±0.00	0.05±0.00*	0.06±0.01*
**PI 34:2**	0.02±0.00	0.04±0.00*	0.05±0.00*
**PI 36:0**	ND	ND	0.004±0.00
**PI 36:1**	0±0.00	0.01±0.00*	0.03±0.00*
**PI 36:2**	0.02±0.00	0.05±0.00*	0.12±0.01^§^*
**PI 36:3**	0.02±0.00	0.03±0.00	0.05±0.00^§^*
**PI 36:4**	0.07±0.01	0.1±0.01	0.18±0.02^§^*
**PI 36:5**	0.00±0.00	0.0005±0.00*	0.005±0.00*
**PI 36:6**	0.00±0.00	0.00±0.00	0.004±0.00*
**PI 38:4**	0.31±0.07	0.38±0.09	0.68±0.12*
**PI 38:5**	0.02±0.00	0.03±0.00	0.06±0.00^§^*
**PI 38:6**	0.01±0.00	0.01±0.00*	0.02±0.00^§^*
**PI 40:3**	0.00±0.00	0.00±0.00	0.01±0.00^§^*
**PI 40:5**	0.00±0.00	0.01±0.00*	0.02±0.00*
**PI 40:6**	0.01±0.00	0.03±0.00*	0.04±0.00*
**PI 40:7**	0.00±0.00	0.006±0.00*	0.008±0.00*

Abbreviation: PI: Phosphatidylinositol

* Significant differences compared with sham group.

^§^ Significant differences compared with 6h I/R groups, *P* < 0.05.

Of the less abundant PL species, namely PE, PS and PG, only total PG concentrations increased significantly following 24h I/R when compared to sham-operated and 6h I/R groups (41.39±6.96 versus 25.78±3.47 and 28.23±2.78 ng/mg of tissue, respectively) (Fig 7D). Of the 16 identified PG species in renal tissue, 9 and 15 PG species increased significantly in 6h and 24h I/R groups respectively compared with sham operated group. PG (34:1) was the most abundant PG, which constituted almost 50% of total PG amounts, with average values representing 15.87±2.20, 14.62±2.82 and 20.64±3.97 ng/mg of tissue in sham-operated, 6h and 24h IR groups, respectively (Table 7).

**Table 7:** Identified PG species in renal I/R.

	Sham (ng/mg of tissue)	6h I/R (ng/mg of tissue)	24h I/R (ng/mg of tissue)
**PG(34:1)**	15.87±2.20	14.62±2.82	20.64±3.97
**PG(34:2)**	2.45±0.40	2.51±0.27	3.23±0.77
**PG(36:1)**	0.86±0.10	0.88±0.04	1.93±0.33^§^*
**PG(36:2)**	2.2±0.30	2.2±0.15	4.2±0.81^§^*
**PG(36:3)**	0.84±0.10	1.54±0.44*	2.05±0.3*
**PG(36:4)**	1.86±0.30	3.46±0.58*	5.41±0.64^§^*
**PG(36:5)**	0.06±0.01	0.08±0.00	0.11±0.01^§^*
**PG(38:4)**	0.45±0.03	0.75±0.08*	1.11±0.18^§^*
**PG(38:5)**	0.28±0.04	0.46±0.07*	0.8±0.04^§^*
**PG(38:6)**	0.4±0.07	0.78±0.17*	0.95±0.03*
**PG(40:4)**	0.01±0.00	0.02±0	0.04±0^§^*
**PG(40:5)**	0.05±0.01	0.09±0*	0.1±0.01*
**PG(40:6)**	0.15±0.02	0.28±0.04*	0.24±0.02*
**PG(40:7)**	0.1±0.00	0.22±0.03*	0.22±0*
**PG(40:8)**	0.12±0.00	0.28±0.1*	0.29±0.03*
**PG(40:9)**	ND	ND	0.01±0^§^*

Abbreviation: PG: Phosphatidylglycerol

* Significant differences compared with sham group.

^§^ Significant differences compared with 6h I/R group, *P* < 0.05.

Despite the appearance of attenuating levels of PE with increasing time of I/R, overall levels remained unchanged (Fig 7E). This apparent attenuation is attributed to the significant reductions in levels of PE(32:0), PE(32:1), PE(34:2) and PE(36:3) that were observed in both 6h and 24h I/R groups compared to sham operated group (Table 8). An additional six PE compounds decreased significantly after 24h I/R, but not 6h. These include PE(36:2), PE(36:4), PE(38:3), PE(38:5), PE(38:6) and PE(38:6e) (Table 8).

**Table 8:** Identified PE species changed significantly in renal I/R.

	Sham (ng/mg of tissue)	6h I/R (ng/mg of tissue)	24h I/R (ng/mg of tissue)
**PE(32:0)**	6.04±0.98	4.12±0.23*	3.17±0.46*
**PE(36:0)**	26.46±6.1	23.32±4.38	20.98±4.48
**PE(32:1)**	2.99±0.32	1.64±0.44*	1.55±0.11*
**PE(34:1)**	17.16±2.45	15.03±2.48	13.39±1.57
**PE(34:1p)**	0.48±0.08	0.45±0.06	0.39±0.14
**PE(34:2)**	32.32±3	23.89±2.45*	21.29±3.92*
**PE(34:2p)**	1.96±0.23	1.91±0.35	1.5±0.3
**PE(36:1)**	11.31±1.99	11.36±0.96	10.9±2.29
**PE(36:2)**	49.16±6.24	42.95±3.23	38.64±4.33*
**PE(36:2e)(36:1p)**	3.93±0.72	3.4±0.6	3.36±0.88
**PE(36:3)**	29.57±5.54	20.55±1.48*	19.78±2.98*
**PE(36:3e)(36:2p)**	4.26±0.93	3.89±0.79	3.86±1.11
**PE(36:4)**	81.17±23.77	61.14±9.85	47.34±6.48*
**PE(36:4e)(36:3p)**	10.82±2.21	9.51±2.12	8.25±2.33
**PE(36:5)**	9.78±2.79	7.66±1.3	6.71±1.06
**PE(36:5e)(36:4p)**	67.48±14.56	54.86±14.37	48.94±13.46
**PE(38:1)**	7.26±1.11	7.74±1.04	7.82±1.89
**PE(38:2)**	2.77±0.39	2.36±0.25	2.47±0.5
**PE(38:3)**	26.85±6.82	19.57±2.85	17.81±1.66
**PE(38:4)**	221.93±61.75	168.53±33.39	155.31±19.3
**PE(38:5)**	66.51±21.43	48.33±7.14	40.02±3.88*
**PE(38:5e)(38:4p)**	58±15.92	51.07±11.41	47.48±10.16
**PE(38:6)**	27.68±8.79	17.27±2.55	16.17±2.07*
**PE(38:6e)(38:5p)**	58.12±9.52	51.2±12.57	36.72±7.45*
**PE(40:1)**	0.58±0.21	0.29±0	0.46±0.15
**PE(40:4)**	4.5±1.54	4.12±0.78	6.01±1.93
**PE(40:5)**	5.01±1.31	4.7±0.48	6.37±1.87
**PE(40:5e)**	4.79±1	4.74±1.27	5.15±1.41
**PE(40:6)**	8.05±2.61	8.51±1.02	10.46±2.77
**PE(40:6e)**	5.78±1.4	6.24±1.72	6.08±1.41
**PE(40:7)**	7.08±1.66	6.2±0.56	7.53±1.29
**PE(40:7e)**	13.77±3.6	13.12±1.98	12.69±2.85
**PE(42:1)**	ND	ND	0.17±0.11
**PE(42:5)**	ND	ND	0.21±0.2
**PE(42:5p)**	0.41±0.12	0.44±0.13	0.57±0.3
**PE(42:6)**	ND	ND	0.24±0.15
**PE(42:6p)**	0.65±0.12	0.79±0.05	0.55±0.3
**PE(42:7)**	0.66±0.41	0.42±0.06	0.64±0.3

Abbreviation: PE: Phosphatidylethanolamine

* Significant differences compared with sham group, P < 0.05.

No significant changes in overall PS concentrations were observed between any of the study groups (Fig 7F); however, some individual PS compounds increased with increased I/R time which are presented in Table 9.

**Table 9:** PS species changed significantly in renal I/R.

	Sham (ng/mg of tissue)	6h I/R (ng/mg of tissue)	24h I/R (ng/mg of tissue)
**PS(32:1)**	0.49±0.06	0.59±0.12	0.83±0.15*^§^
**PS(34:0)**	ND	ND	4.96±1.08*^§^
**PS(34:1)**	14.92±1.3	23.15±4.51*	22.4±4.98
**PS(34:2)**	5.11±0.17	5.34±0.96	10.27±4.02*^§^
**PS(36:0)**	ND	ND	10.44±1.55*^§^
**PS(36:1)**	53.47±5.16	87.88±11.53*	82.17±10.12*
**PS(36:2)**	35.47±3.03	46.32±6.03	62.84±13.93*
**PS(38:1)**	5.86±1.14	6.84±1.23	6.27±1.61*
**PS(38:6)**	36.4±8.48	41.85±7.78	28.23±8.30*^§^
**PS(40:3)**	2.30±0.46	3.37±0.80	3.66±0.52
**PS(40:4)**	3.70±0.83	4.78±0.63	4.72±0.62*
**PS(40:5)**	45.26±10.87	57.27±9.01	44.28±8.01*
**PS(40:6)**	180.04±46.35	219.12±39.28	157.71±32.75*^§^

Abbreviation: PS: Phosphatidylserine

* Significant differences compared with sham group.

^§^ Significant differences compared with 6h I/R group, *P* < 0.05.

## Discussion

Our study identified bioactive fragmented OxPC during renal I/R in an *in vivo* model of renal I/R injury. We found significant increments in fragmented OxPC molecules after 6 and 24h of reperfusion. OxPC levels were also significantly positively correlated with creatinine levels during the reperfusion period. In this study, we identified fifty five OxPC species involving fragmented aldehyde and carboxylic acid containing OxPC derivatives, and also non-fragmented OxPC compounds with terminal furans, isoprostanes and long-chain products in renal I/R. Interestingly, we found that fragmented OxPC, including fragmented aldehyde and carboxylic acid containing OxPC, were produced at greater levels following 24h reperfusion compared to sham operated and 6h I/R groups. Fragmented aldehyde containing OxPC increased significantly as early as 6h reperfusion. Their levels increased by increasing time of reperfusion, as there were significant differences between 24h I/R groups compared to 6h I/R and sham operated groups. Fragmented carboxylic acid containing OxPC were produced later in renal I/R, as significant elevations in their levels were observed only after 24h I/R, but not 6h. In the current study, both fragmented aldehyde and carboxylic acid containing OxPC levels were significantly correlated with severity of I/R injury as measured by creatinine levels. The three most abundant fragmented OxPC in rat kidney were PazPC, SONPC and PONPC. Similar to our results, Lloberas et al., (2002) showed that OxPLs with platelet-activating factor (PAF) activity (PAF-like lipids), are produced following 15 min of reperfusion in rat kidney. Phospholipid oxidation has also been implicated in other renal disease models. In patients with lecithin:cholesterol acyltransferase (LCAT) deficiency, renal tissue displayed increased OxLDL antibody binding to the glomeruli, indicating OxLDL epitopes increased in these patients [29]. Also fragmented OxPC species are potent inducers of cell death in smooth muscle cells, macrophages, oligodendrocytes, and endothelial cells [30–32]. Previous data from our laboratory demonstrates that exogenous administration of POVPC, PONPC, PGPC, and PAzPC to postnatal cardiac cells triggers cell death in a dose dependent manner [33]. Additionally, fragmented OxPC species, namely POVPC and PGPC, increased significantly following ischemia and I/R in adult rat cardiomyocytes. Ganguly et al.,(2017) further demonstrated that in cardiomyocytes exposed to fragmented OxPC, specifically PGPC and PONPC, apoptotic pathways are activated, resulting in cell death [25].

In the current study, despite a significant increase in non-fragmented isoprostane levels after 6h I/R, a significant decrease was observed after 24h reperfusion. Isoprostanes are formed via non-enzymatic oxidation from arachidonic acid and exert prostaglandin-like properties. Unlike enzymatic oxidation, non-enzymatic oxidation, which occurs during I/R, is not site-specific. Therefore, oxidative stress may result in the production of OxPL with anti-inflammatory properties; however, most OxPLs have potent inflammatory effects [34]. Cyclopentenone containing OxPLs like 1-palmitoyl-2-epoxyisoprostane-sn-glycero-3-PC (PEIPC) and 15-deoxy-Δ12,14-prostaglandin J2 share similar structures. Both 15-deoxy-Δ12,14-prostaglandin J2 and cyclopentenone containing OxPLs can block NFκB- dependent inflammatory responses and activate NF-E2-related factor 2 (Nrf2), which is a transcriptional regulator of the antioxidant response [35]. In a study by Bretscher et al., (2015), administration of cyclopentenone OxPCs to myeloid cells inhibited expression of proinflammatory cytokines and chemokines via activation of Nrf2 [35]. Moreover, they also showed the protective effects of cyclopentenone OxPC against sepsis-associated lung injury [35]. As previously mentioned, isoprostanes increased following 6h, but not 24h, I/R. AKI severity does not appear to be dependent upon the presence of isoprostanes or other non-fragmented OxPC molecules as suggested by the poor correlation of these compounds in renal tissue with plasma creatinine levels. It should be considered that the potential increase in renal OxPC could be underestimated given that isoflurane was used as anesthetic agent. It has been previously shown that volatile anesthetics can result in renal protection during I/R [36].

Membrane phospholipid concentrations in renal tissue following I/R injury were also assessed in our three study groups. Significant increases in PC, SM, PI and PG were observed after 24 I/R. PC represented the most abundant PL in kidney tissue for all study groups and is the precursor of OxPC compounds. Surprisingly, total PC concentrations were unchanged after 6h I/R, despite increases in OxPC levels at the same time point. In addition, 24h I/R resulted in significant increases in both PC and OxPC molecules. Rao et al., (2016), has demonstrated elevations in PC levels in a mouse model of renal I/R involving 12 mice after 30 min renal ischemia followed by 6h and 24h reperfusion. In this non-targeted lipidomic analysis, 52 PC species increased significantly after 24h reperfusion [37]. Similar to our results, they observed significant elevations in PC(34:1), PC(36:1), PC(36:2), PC(38:2), PC(38:4), PC(38:5), PC(40:2) and PC(40:4) following 24h reperfusion. Moreover, choline incorporation into PL is accelerated during renal ischemia and I/R particularly in the proximal tubule [38–40], which may result in more PC biosynthesis in renal I/R.

Phospholipase A2 is activated in renal I/R. Phospholipase A2 hydrolyses the fatty acid at the Sn-2 position of PLs resulting in elevations of free fatty acid and lyso phospholipids [41]. In the current study, LPC (22:5) was the only lyso PC species that increased significantly after 6h I/R, but was normalized after 24h. Rao et al., (2016) reported significant elevations in lyso PC (18:0) and (20:4) levels after 24h reperfusion [37]. Similarly, Liu et al., (2012) observed significant increases in three LPC species in a rat model of kidney I/R, namely 1-stearoylglycerophosphocholine (18:0), oleoyl-glycerophosphocholine (18:1) and palmitoyl-glycerophosphocholine (16:0) after 24h I/R [3]. Wie et al., (2014) also evaluated metabolomic changes in renal I/R and determined that levels of 2-palmitoylglycerophosphocholine (16:0), decreased significantly in plasma after 2h I/R and normalized after 7 days [42]. Given that LPC are breakdown products of membrane phospholipids, it was suggested that membrane breakdown and remodeling activity might be transiently inhibited early after renal I/R.

SM is a membrane PL composed of a choline head group and ceramide. In the current study, total SM concentrations increased only after 24h I/R compared to both the sham and 6h I/R groups. No changes were observed after 6h I/R relative to sham. Rao et al., (2016) reported elevations in SM concentrations in 34 of 40 identified SM species after 24h I/R [37]. Wie et al., (2014) [42] observed elevations in palmitoyl sphingomyelin and stearoyl sphingomyelin, which are the hydrolyzed form of SM, post 48h I/R. In addition, the present study measured significant increases in total concentrations of PI and PG after 24h I/R. Rao et al., (2016) [37] and Liu et al., (2012) [3] did not report PI and PG levels. No other literature to our knowledge has identified specific PI and PG molecules in renal tissue from rats undergoing 6 and 24h I/R. Despite a non-significant decreasing trend in overall PE compounds with increased I/R time, 10 PE molecules significantly decreased in both I/R groups compared to sham. These results agree with Rao et al., (2016) who observed significant reductions in PE compounds after 24h reperfusion [37]. There are two distinct forms of cytosolic PLA2 in rat kidneys that are activated following reperfusion. Unlike the large molecular weight form, which is active against both PE and PC, the small molecular weight form of PLA2 has activity only against PE [43]. This could be a potential mechanism towards a decreased trend in PE levels in our study.

Previous studies report accumulation of lipids in various renal diseases. In diabetic nephropathy, lipid accumulation occurs due to increases in glomerular filtration of protein bound lipids, which results in proteinuria [44]. Evidence from *in vitro* and *in vivo* renal I/R injury studies has also revealed that accumulation of triglycerides and cholesterol can occur [38, 39, 45].

It has also been suggested that renal cells, contrary to cardiac or brain cells, can recover from injury even after prolonged ischemia through various intrinsic, extrinsic mechanisms and also paracrine influences [37]. Increased levels of growth factors IGF-1[38, 39], fibroblast growth factors [40–42], and hepatocyte growth factor [43, 44] have been reported in post ischemic kidney which all can lead to repair processes. Few hours after I/R injury, proliferation of surviving tubular epithelial cells (TECs), which contain phospholipids in their structure [40], begins which eventually results in kidney damage recovery [40]. Wan et al., 2015 showed that proliferation and differentiation of surviving TECs begins at 24h after kidney IR injury [46]. In addition, abnormal repair may occur which results to renal fibrosis and then chronic kidney disease. Due to abnormalities in glomerular filtration, lipoproteins which contain phospholipids may **accumulate** in the kidney [46].

Membrane polarity can also change following changes in membrane phospholipids and their oxidised form. Lipid polarity of apical and basolateral membranes of renal epithelial cells are vastly different. The apical membrane of rat kidney is rich in sphingomyelin, phosphatidylserine and a high ratio of cholesterol-to-phospholipid. However, phosphatidylcholine and phosphatidylinositol are prevalent in the basolateral membrane [47]. It has been shown that following ischemia, ratios of sphingomyelin-to-phosphatidylcholine and the cholesterol-to-phospholipid decrease significantly which result in partial loss of surface membrane polarity [48]. Bruce et al., (1988) also assessed surface membrane polarity following 15 min of ischemia and 2 hr of reperfusion. They isolated apical membrane fractions and found that both apical NaK-ATPase specific activity and enrichment increased following 15 min of ischemia. They also observed a partial loss of apical lipid polarity, which was accompanied by significant reductions in ratios of sphingomyelin to phosphatidylcholine and cholesterol to phospholipid. They suggested that apical and basolateral membrane lipids move to the alternate surface membrane domain during ischemic injury which is the reason of marked reduction in the SM/PC ratio in apical membrane fractions[48]. On the other hand, OxPL may also alter the properties of biological membranes, since they have different polarity and shape compared with their non-oxidised molecules [49].

## Conclusions

To conclude, we have shown for the first time that bioactive OxPC species are produced during renal I/R, with levels increasing with increased reperfusion time. Fragmented OxPC, including both aldehyde and carboxylic acid molecules, had the strongest positive correlation related to AKI. These compounds may serve as potential therapeutic targets for mitigating the progression of oxidative injury in studies involving renal I/R. We can utilize the potential attenuation of OxPC activity to reduce renal I/R injury. One potential agent is E06 which is a monoclonal IgM antibody that specifically binds to fragmented OxPCs. E06 has been used in LDL -/- animals to inhibit OxPC activity resulting in 46% reduction in atherosclerotic lesion formation [50, 51].

## Supporting information

S1 Table MRM product ions for PS species. (DOCX)
