# Supplementary material for: Oxidized phosphatidylcholines are produced in renal ischemia reperfusion injury
Source: PLoS One. 2018 Apr 23;13(4):e0195172. doi: 10.1371/journal.pone.0195172 (PMC5912739; doi:10.1371/journal.pone.0195172)
Supplement: S1 Table — (DOCX) [file pone.0195172.s001.docx]

**S Table 1: MRM product ions for PS species**

| **Species** | **Q3 mass (Da)** |
| --- | --- |
| PS(32:0 std) | 647.5 |
| PS(32:1) | 645.5 |
| PS(34:0) | 675.5 |
| PS(34:1) | 673.5 |
| PS(34:2) | 671.5 |
| PS(36:0) | 703.6 |
| PS(36:1) | 701.6 |
| PS(36:2) | 699.5 |
| PS(36:3) | 697.5 |
| PS(36:4) | 695.5 |
| PS(38:1) | 729.6 |
| PS(38:2) | 727.6 |
| PS(38:3) | 725.6 |
| PS(38:4) | 723.5 |
| PS(38:5) | 721.5 |
| PS(38:6) | 719.5 |
| PS(40:3) | 753.6 |
| PS(40:4) | 751.6 |
| PS(40:5) | 749.6 |
| PS(40:6) | 747.5 |
| PS(40:7) | 745.5 |
